# Intelligent Prediction of Fan Rotation Stall in Power Plants Based on Pressure Sensor Data Measured *In-Situ*

**DOI:** 10.3390/s140508794

**Published:** 2014-05-19

**Authors:** Xiaogang Xu, Songling Wang, Jinlian Liu, Xinyu Liu

**Affiliations:** 1. School of Energy Power and Mechanical Engineering, North China Electric Power University, 619 Yonghua North Street, Baoding, 071003, China; E-Mails: hdwangsl@163.com (S.W.); ljl_gongzuo@163.com (J.L.); 2. Department of Mechanical Engineering, McGill University, 817 Sherbrooke Street West, Montreal, Quebec H3A 0C3, Canada; E-Mail: xinyu.liu@mcgill.ca

**Keywords:** rotation stall prediction, support vector machine, wavelet transform, energy efficiency

## Abstract

Blower and exhaust fans consume over 30% of electricity in a thermal power plant, and faults of these fans due to rotation stalls are one of the most frequent reasons for power plant outage failures. To accurately predict the occurrence of fan rotation stalls, we propose a support vector regression machine (SVRM) model that predicts the fan internal pressures during operation, leaving ample time for rotation stall detection. We train the SVRM model using experimental data samples, and perform pressure data prediction using the trained SVRM model. To prove the feasibility of using the SVRM model for rotation stall prediction, we further process the predicted pressure data via wavelet-transform-based stall detection. By comparison of the detection results from the predicted and measured pressure data, we demonstrate that the SVRM model can accurately predict the fan pressure and guarantee reliable stall detection with a time advance of up to 0.0625 s. This superior pressure data prediction capability leaves significant time for effective control and prevention of fan rotation stall faults. This model has great potential for use in intelligent fan systems with stall prevention capability, which will ensure safe operation and improve the energy efficiency of power plants.

## 1. Introduction

The fan is a common type of air flow control component, widely used in many impeller machines, such as ventilators, compressors, and pumps. These machines are employed in almost every field of industry and consume a significant amount of electricity. In thermal power plants, fans are the power sources of smoke exhaust systems, and blowing fans and induced draft fans of boilers account for approximately 30% of the plant electric consumption [1]. Thus, the fans in smoke exhaust systems should be targeted as an important candidate machinery for energy saving and emission reduction. The operation state of fans in the smoke exhaust systems also has significant effects on the safe and economic operation of a power plant.

According to the statistics of the Electric Power Research Institute (EPRI), 30%∼50% of all power plant outage failures are caused by rotating machinery, such as steam turbine generators, fans and pumps. Based on the Chinese statistics of power plant operations in 2007, blower fan and induced draft fan failures are the second frequent cause of unscheduled shutdowns of thermal power plants, which causes significant economic losses. Therefore, effective approaches for online diagnosis and active control of common faults in power plant fans are urgently needed to ensure the reliable operation of the plant. Avoiding operations of the fans in their close-to-fault modes will also improve the energy efficiency of the impeller machinery.

Rotation stalls are the most common cause of fan faults. One or several stall cells will form in the fan impeller passage when the flow of impeller machinery reduces to a certain limit. The occurrence of rotation stalls changes the stable flow field in the impeller, generates additional loads, and can even cause fatigue and fracture of the blades. If the rotation stall cannot be effectively contained, a flow surge may occur and lead to decreased unit efficiency and even vibrations of the fan body and connection pipes, which could possibly cause damages. Therefore, particular attention should be paid to the weak-stall mode of fans during operation, and active prediction and subsequent control of early-stage rotation stall in fans should be pursued.

The mechanism and detection of fan rotation stall have been actively studied in the industrial fan and compressor community. The mechanism of rotation stall has been investigated through signal processing methods or experimental approaches. For instance, Longley *et al.* [2] used spatial Fourier analysis of pressure signals in order to detect changes in the amplitude and phase of modal wave. McDougall *et al.* detected the transient process of rotation stalls through detailed measurements [3]. The continuous wavelet transform method was also introduced by Lin *et al.* for signal analysis of compressor stalls [4,5], allowing the analysis of the disturbance propagation process in the rotation stall development process of axial flow compressors.

For rotation stall detection, Cameron and Morris [6] proposed a cross-correlation method of circumferential space sensor signals, which effectively detected the abrupt onset of rotation stalls. Bianchi *et al.* [7] developed a stall detection system for induced draft fans of coal-fired power plants, in which a new methodology was proposed for the early detection of stalls in low-speed axial-flow fans used for tunnel ventilation. Teolis *et al.* [8] presented algorithms to detect stalls for gas turbine engines using data from eddy current sensors. Li *et al.* [5] enhanced the characteristics of unsteady rotor tip flows with the help of time- and scale-averaged wavelet spectra of a high-speed, single-stage compressor and a low-speed, three-stage compressor. Sheard *et al.* [9] presented a methodology involving a symmetrized dot pattern (SDP) technique that is capable of differentiating between stall conditions that constitute a mechanical risk and those that do not. Shiomi *et al.* [10] carried out an experiment in a semi-open propeller fan using a hot-wire anemometer, and two types of different periodical fluctuations whose frequencies were not the same as the rotor rotation frequency were found in the relatively high flow-rate region and low flow-rate region.

Most of the aforementioned research work targeted rotation stalls of the compressors of aero-engines, studied the unsteady characteristics of rotation stalls, and explored approaches for expanding the steady state operation and detecting stalls online. However, compared with the axial flow compressors, research on the rotation stalls of centrifugal fans is limited. There are significant differences in the rotation stall mechanisms between centrifugal fans and axial flow compressors [11]. More importantly, most techniques existing in the literature were focused on detecting the starting point of the rotation stall and cannot predict, ahead of a certain time period, the actual stall occurrence. This limits the application of these techniques in active control of fans to avoid rotation stalls. Detecting instability signs in the process of fan operations as early as possible and taking appropriate measures to prevent fan stalls are important for improving the efficient operation and avoiding unnecessary breakdowns of power plants. In addition, rotation stall prediction will also improve the energy efficiency of the fans, improve the efficiency of the thermal power plants, and thus reduce their CO_2_ emissions.

This article presents a novel intelligent model, based on support vector regression machine (SVRM), for predicting the rotation stalls of centrifugal fans from pressure data measured online. Many experiments are performed under different running conditions on a centrifugal fan setup to obtain pressure signals of the fan operation process from normal state to rotation stall. The frequency characteristics of the pressure signals before and during rotation stalls are first analyzed using Fourier transform, and the pressure signals are then used to train the SVRM prediction model. The trained prediction model can accurately predict the fan pressure data 0.0625 s in advance, based on which the rotation stalls can be reliably detected. This model allows the active control of rotation stalls before their occurrence, which represents a major advantage over existing stall prediction techniques.

## 2. Experimental Setup of a Centrifugal Fan System

**Figure 1.**
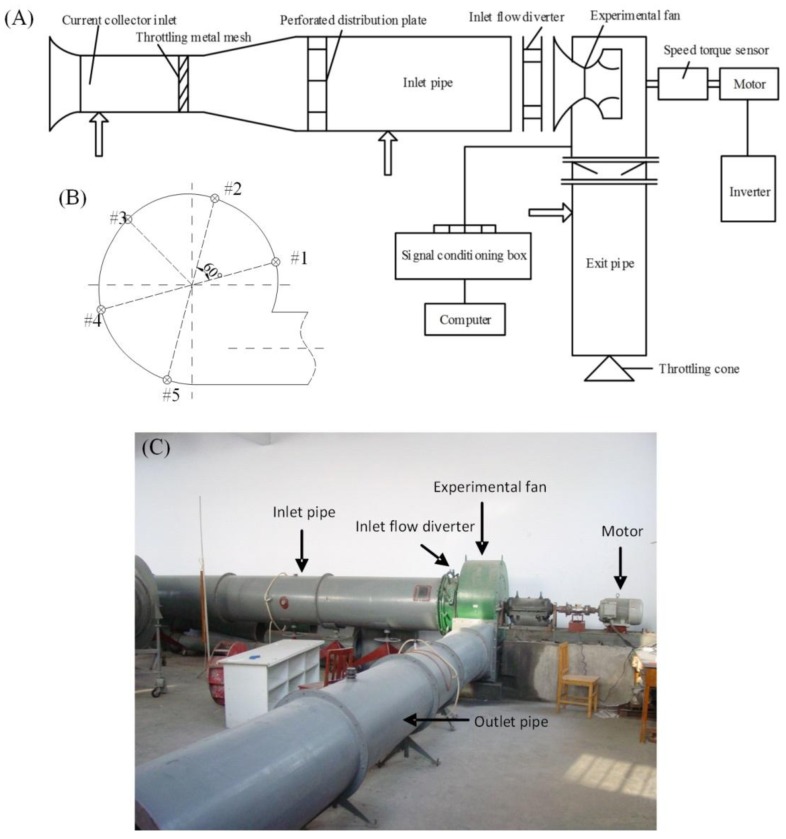
Experimental setup for internal pressure measurements of a centrifugal fan operated at normal conditions and under rotating stalls.

The experiments were conducted on the 4-73No8D centrifugal fan, which is a common model from the 4–73 series widely used in Chinese thermal power plants. The experimental setup, as schematically shown in Figure 1A, consists of the centrifugal fan (nominal discharge coefficient: q̄_v_ = 0.205; accuracy of speed adjustment: 0.3 rotations) attached to an alternating-current (AC) motor, inlet and outlet pipelines connected to the fan, an inlet flow divider between the inlet pipeline and the fan, and a signal conditioning unit connected with a computer. Five piezoresistive pressure sensors (measurement range: 20 kPa; accuracy: 10 Pa) were arranged on the inner surface of the fan casing with an angular spacing of 60° (Figure 1B), to measure the aerodynamic pressures of the air flow inside the fan. The pressure signals were recorded during the fan operation from a normal state to a rotation stall state, with a rotational speed of 1300 rpm. The experiments were repeated four times when the openings of the guide vanes were set to be 0°, 15°, 30° and 45°. The transition of the fan from the normal state to the rotation stall state was controlled by adjusting the resistance of the pipe network using a throttling cone installed in the outlet pipe. A photograph of the experimental setup is shown in Figure 1C. The signal conditioning unit (9118DG; ADLINK Technology, Taipei, Taiwan) was used to simultaneously acquire the pressure signals from the five pressure sensors at 320 Hz.

## 3. Characteristic Analysis of Pressure Signals from Fan Normal Operation and Rotation Stalls

### 3.1. Fourier Transform Frequency-Domain Analysis of Pressure Data

For pressure data analysis, the Fourier transform method was first adopted to extract the frequency distribution, the fundamental frequency, and the harmonic frequencies of the pressure data obtained in normal operations and fan rotation stall conditions. These results helped us understand the frequency characteristics of the fan rotation stalls. However, the Fourier transform results cannot reflect the gradual development of fan rotation stall, and thus cannot be used for rotation stall prediction. In recent years, with the advances in unsteady flow research, wavelet analysis has been applied to the time-frequency characteristic analysis of the mechanically unsteady flows of impellers. In this research, we applied the wavelet analysis to decipher the time-frequency characteristics of the pressure signals obtained during the transition process from normal operation to rotation stall, to quantitatively reveal the gradual development of rotation stalls, and to formulate a novel method for predicting the onset of rotation stalls.

**Figure 2.**
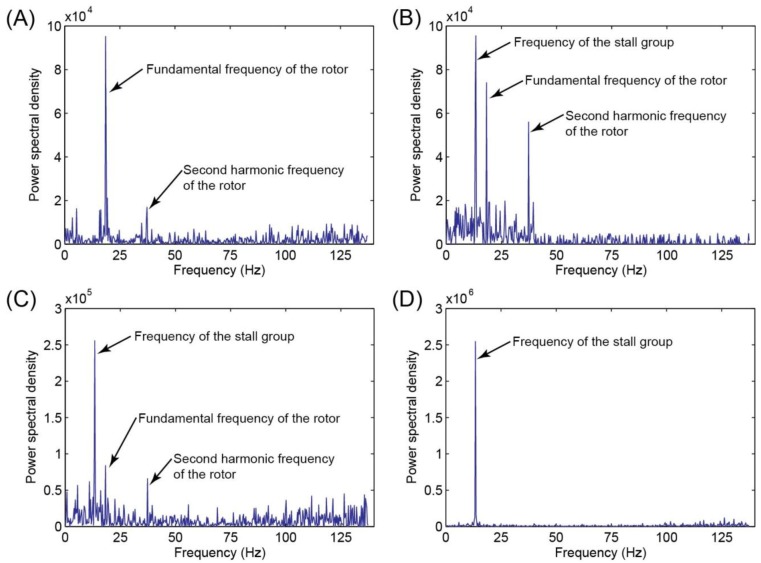
The spectrum diagram of pressure signal (from sensor #2) in the development of a rotation stall. From (**A**) to (**D**), the flow rate was gradually decreased by adjusting the throttling cone, corresponding a discharge coefficient (q̄_v_) of 0.172, 0.154, 0.151, 0.147, respectively. Starting from (**B**), the rotation stall occurred and developed from a weak stall in (**B**) into a strong stall cell in (**C**) and (**D**).

The signals from all the five pressure sensors were analyzed using fast Fourier transform (FFT), and the FFT spectra were used to: (i) quantify the fundamental frequencies of the fan rotor and the rotating stall group, and (ii) reveal the graduate development of the stall group. Using the experiment with a full opening (0°) of the guide vane as an example, Figure 2 shows the FFT spectra of the pressure data from sensor #2 (Figure 1B) at different stages of the stall development. Pressure data obtained in 3.125 s (1000 data points) were used for FFT.

From Figure 2, one can make the follow observations; (1) Under the normal operation conditions, the spectra of the pressure signal mainly included the rotational (fundamental) and harmonic frequencies of the rotor; (2) With the decrease of the flow rate (indicated by *q̄_v_*), the fan deviated from the normal operating conditions into the stall region. As shown in Figure 2B, the weak stall group appeared in the spectra at 14.4 Hz (approximately 2/3 of the fan fundamental frequency of 21.7 Hz); (3) The weak stall regime corresponds to the range of low flow rates with small perturbations. With the further development of the rotation stall from the weak regime (Figure 2B) to the strong regime (Figure 2C,D), the magnitude (*i.e.*, energy) of the stall group component increased substantially. During this transition, the flow perturbations, reflected in the pressure signals, were also magnified significantly.

### 3.2. Time-Frequency Analysis of Pressure Data Using Daubechies Orthogonal Wavelet

Through Fourier transform of the pressure signals, we have clearly observed the gradual development of the rotation stall and its frequency characteristics. However, the Fourier transform cannot reveal the time-domain characteristics of rotation stalls, and thus cannot be used for stall prediction. Differently, wavelet transform can identify signal components with different frequencies and the time associated with these frequencies, and has been widely applied to analysis of non-stationary signals [12]. The wavelet transform can decompose a time-domain signal into basis functions which are known as wavelets, and the decomposition process is computed separately for a series of frequency sub-domains, representing a multi-resolution analysis capability. To apply wavelet transform to signal analysis, we chose as the wavelet basis function the Daubechies orthogonal wavelet, which has the advantages of compact support, regularity, multi-resolution analysis ability, and high sensitivity to non-stationary signals. Daubechies wavelets are a family of orthogonal wavelets for discrete wavelet transform with a maximum number of vanishing moments [13].

In this work, the Daubechies orthogonal wavelet was utilized for multi-resolution decomposition of fan pressure signals and obtaining of the wavelet coefficients of the relevant frequency bands, which were used for detecting the rotation stall from the pressure data. The pressure signal (denoted by *S*) was decomposed, using Daubechies orthogonal wavelet, into the *N*^th^-level approximation component (denoted by *A_N_*, where *N* is the number of wavelet layers) and *N* number of detail components (denoted by *D_i_*, *i* = 1, 2, …, *N*) covering different frequency bands. The components *A_N_* and *D_N_* decomposed at different scales have different resolutions of time and frequency. The decomposition equation can be expressed by *S* = *A_N_* + *D_N_* + … + *D*_2_ + *D*_1_. Changes in the frequency and amplitude of the pressure signal during fan operation were examined by analyzing a specific detail component *D_j_* with a frequency band containing the rotation stall frequency of the pressure signal. Daubechies orthogonal wavelet is generally denoted by db*N* where *N* = 1, 2, …, 10, and the effective support length of the wavelet function and scale function of db*N* is 2*N*−1. The vanishing moment order of the wavelet function is *N*. The bigger the value of *N* used, the longer and smoother corresponding wavelet will be. We selected the common db4 wavelet to analyze the dynamic pressure signals of the fan, which was proved, through decomposition trials, to be sufficient for the current application.

**Figure 3.**
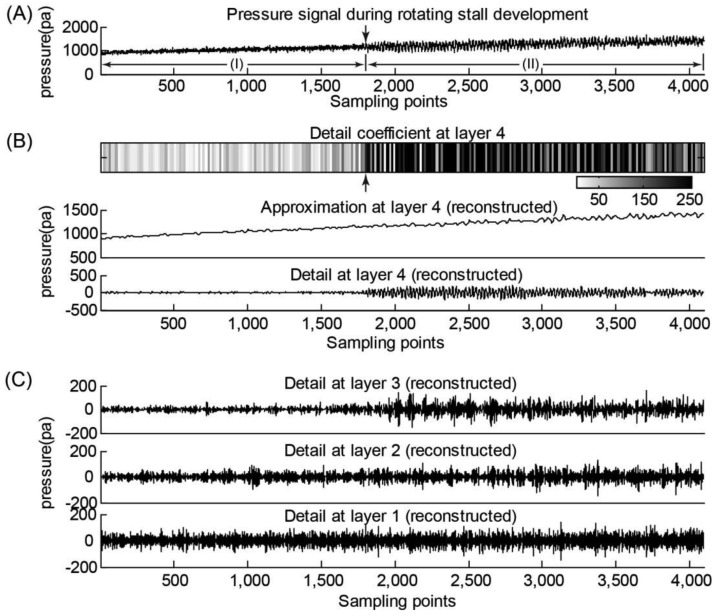
Fan casing pressure signal (from sensor #2) and its time-frequency map of wavelet analysis when βbeta; βequals; 15βdeg;. (**A**) Original pressure signal. (**B**) Decomposed detail component of the pressure signal at layer 4 (10–20 Hz). The detail coefficient at layer 4 indicates the energy of the pressure component caused by the fan rotation stall. (**C**) The decomposed detail components of the pressure signal at layers 1–3.

The pressure signals during the gradual development of fan rotation stall were obtained at 1300 RPM, and the opening of the guide vane was set to be 0°, 15°, 30° and 45°. The wavelet transform results of the pressure data from sensor #2 (guide vane opening: 15°), obtained during the rotating stall development, are shown in Figure 3. Daubechies wavelet transform of pressure signals from other sensors showed similar trends during rotation stall development. Because the sampling frequency was 320 Hz, the wavelet analysis frequency was set to be 160 Hz. The frequency band of the detail coefficient of the third layer (or third-layer detail coefficient) is 20–40 Hz, and the rotational frequency (21.7 Hz) of the fan lies in this band. The frequency band of the detail coefficient of the fourth layer (or fourth-layer detail coefficient) is 10–20 Hz, and the rotating stall frequency (14.4 Hz; obtained from the FFT analysis in Section 3.1) of the fan lies in this band.

Thus, we used the four-layer detail coefficient as a quantitative indicator for detecting the onset of a rotation stall. For the pressure data from sensor #2 (guide vane opening: 15°), we empirically set a threshold of 200 Pa for the fourth-layer detail coefficient, and any pressure data point yielding a four-layer detail coefficient above 200 Pa indicates the presence of the rotation stall. This empirically-chose threshold was proven to be robust for other pressure data sets collected from sensor #2 at the guide vane opening of 15°. In Figure 3A, the arrow-labelled pressure data point (t = 5.6 s) was identified as the starting point of the rotation stall. One can observe that, during the development of rotation stalls, the internal pressure of the fan casing constantly increased with the gradual decrease of the flow rate and some decentralized energy components gradually emerged in the fourth-layer frequency band of the pressure signal, reflecting the onset of rotation stall. Two ranges (I) and (II) labelled in Figure 3A refers to the normal operation state and rotation stall stage, respectively.

## 4. The SVRM Model for Predicting Pressure Data in the Development of Fan Rotation Stalls

At the initial stage of fan rotation stall, the formed stall groups are not stable and tend to circularly occur, rupture, disappear, and re-develop with changes of the flow field [11]. If the onsite of a rotation stall can be accurately predicted in advance, active control methods can be used to adjust the flow field conditions, thus avoiding the occurrence of rotation stalls, and guaranteeing the stable working operation of the fan [11,14].

To effectively predict the fan rotation stall, we proposed a support vector regression machine (SVRM) model for multi-step prediction of the pressure data. These predicted data were then used to predict the fan rotation stall via wavelet transform. For initial training of the SVRM model, sample data of the pressure signals were obtained during the transition process from normal operation to rotation stall. Since the SVM model can predict the pressure data of the fan by a certain time advance, the rotation stall can then be *predicted* rather than detected. This work was focused on the theoretical development and experimental validation of the SVRM prediction model, and the follow-up fan active control will be pursued in our future research.

The proper selection of a suitable prediction model is the key to ensure the precision and robustness of the rotation stall prediction. Existing regression models for data prediction mainly include support vector machines [15,16], neural networks [17,18], chaos theory [19,20], and so on. The SVRM has advantages over other prediction models in the following aspects: SVRM is a supervised learning method particularly powerful in addressing pattern recognition problems with small sample sizes, nonlinearity, and high dimensions. It has also been applied to function curve fitting and other machine learning problems. In comparison to neural networks, SVRMs have better generalizability in regression. Additionally, SVRMs dissociate the number of input vectors and the computational complexity. Moreover, the solution of SVRM models can be converted into the solution of convex optimization problems, which guarantees the global optimality of the algorithm and avoids local-minimum traps commonly existing in the neural network models.

For the training of SVRMs, the feature extraction and selection are crucial tasks. Feature extraction is difficult in many prediction applications, and feature selection is largely subjective and usually induces lack or redundancy of information. If we simply take the time series as the input vector for SVRM training, the input vector contains less information and will result in low accuracy of prediction. Therefore, we employed a phase space transformation approach based on chaos theory for preprocessing the time series and reconstructing their phase space which was then used as the input vector for SVRM training.

The chaos characteristics of the pressure data were first analyzed using chaos theory [19,21]. The SVRM prediction model was then set up by following steps: (1) To determine the input dimension and the training data set for the SVRM model. The embedding dimension and delay time are critical parameters to the phase space transformation, which were carefully selected to reconstruct the phase space of the time series; (2) To train the SVRM model using the training sample data selected in the step 1. In order to verify the accuracy and generalizability of the prediction model, the pressure signals of the rotating stall gradual process were taken as the training data when the opening of guide vane was 15°, and the pressure signals under the other openings of the guide vane were taken as the testing data.

### 4.1. The Phase Space Reconstruction of Pressure Signal

The evolution of any signal in a system is determined by its interactions with other signal components. Thus, the evolution of one signal includes the information of other related signals. The characteristic of the system can be extracted from a set of time series data of any signal in the system, and this characteristic can be represented by a trajectory in a high-dimensional space. Takens [22] proved that a space can be found to restore the original characteristic of the system using chaos theory.

Using *x*(1), *x*(2), …, x(*n*) to denote a set of pressure data measured during the rotating stall development, the reconstructed phase space of the pressure signal obtained can be expressed by:

(1) $$\{\begin{array}{c} X_{1}=[x(1),x(1+\tau ),\cdots ,x(1+(m-1)\tau )]\\ X_{2}=[x(2),x(2+\tau ),\cdots ,x(2+(m-1)\tau )]\\ \vdots \\ X_{M}=[x(M),x(M+\tau ),\cdots ,x(M+(m-1)\tau )] \end{array}$$

where *M* is the dimension of the pressure signal phase space: *M* = *n* – (*m* – 1)*τ*, *m* is the embedding dimension, and *τ* is the time delay. The training input for the prediction model is then *X* = [*X*_1_, *X*_2_, …, *X_M_*] and the training output is Y = [*x*(2 + (m − 1) *τ*), …, *x*(*n*)].

The selection of the *delay time* and the *embedding dimension* is vital to successful phase space reconstruction of the pressure data. We employed the popular autocorrelation function method [23] to determine the delay time to be *τ* = 2 s, which was defined by the time corresponding to the minimal data points. To select an effective embedding dimension, we used the Cao method [24] which can effectively distinguish the difference between the random signals and deterministic signals. The embedding dimension was determined to be *m* = 3.

### 4.2. Training of the SVRM Prediction Model

Using the reconstructed phase space of the pressure time series (*X* = [*X*_1_, *X*_2_, …, *X_M_*]) as the input space, the training of the SVRM model becomes an optimization problem to find a function of the input vector, *Y*(*X*), which can accurately calculate (or predict) the next pressure sampling point *y* based on the reconstructed phase space of the previously measured pressure time series *X_i_*. Based on the SVR theory, the function *Y*(*X*) has a general linear format:

(2) $$Y(X)=(w^{T}\varphi (X)+b)$$

where *φ* is a kernel function, *w* is a weighing vector with the same dimension as *φ*, and *b* is a bias.

The function *Y*(*X*) is determined by solving the following optimization problem:

(3) $$\underset{w,b,\xi }{\min}\frac{1}{2}w^{T}w+C\sum_{i=1}\xi_{l}$$

subject to *y_i_*(*w^T^ϕ*(*x_i_*) + *b*) ≥1−*ξ_i_*, where *ξ_i_* ≥ 0.

Here, training vectors *X_i_* are mapped into a higher dimensional space by the kernel function *φ*. SVRM finds a linear separating hyperplane with the maximal margin in this higher dimensional space. For the kernel function, we chose the widely used radial basis function (RBF) kernel:

(4) $$\begin{array}{ll} \phi (x_{i},x_{j})={\left(\gamma x_{i}^{T}x_{j}+r\right)}^{d}, & \gamma >0 \end{array}$$

Accurate determination of the parameters *C* and *γ* is critical to guarantee the regression effectiveness. We performed a grid search to find out the values of *C* and *γ*. Different combinations of (*C*, *γ*) values were iterated and the one yielding the best regression accuracy was selected. Exponentially growing sequences of *C* and *γ* (*i.e.*, 2^−10^, 2^−9^, … , 2^10^) were used for iteration. To reduce the computation complexity, we first performed a coarse grid search to identify a region including the optimal combination of *C* and *γ*, and then conducted a fine grid search in that region. After *C* and *γ* were found, the parameters *w*, *b*, and *ξ* were calculated from Equation (3) and the function *Y*(*X*) (Equation (2)) was determined.

## 5. Experimental Validation of the SVRM Model for Rotation Stall Prediction

After being trained using the sample pressure data, the SVRM model is ready for rotation stall prediction. The major advantage of this SVRM prediction model is that it can provide accurately predicted pressure data ahead of a certain time, which enables *prediction* rather than detection of rotation stalls. This model can be combined with some popular time-domain stall detection techniques [25,26] for online stall prediction (that is, the SVRM model predicts pressure data and the stall detection technique determines the starting time point of the emerging stall). The scope of this paper is to establish the SVRM prediction model and validate the feasibility of using this SVRM model for rotation stall prediction. Thus, we opted to use offline wavelet transform as the stall detection method to detect stalls from both the measured and the predicted pressure data. As we will present in this section, the stall detection results (*i.e.*, starting point of the stall), based on both measured and predicted data sets, agree well with each other. This proves the feasibility of using this SVRM model for rotation stall prediction. To achieve online stall prediction, one could adopt these time-domain stall detection techniques [25,26] to further process the predicted pressure data, in real time (computation time shown in Table 1), using the SVRM model.

With a fixed sampling frequency, the time advance by which the rotation stall can be predicted mainly depends on the number of prediction steps performed by the SVRM model. We performed pressure data prediction in single and multiple steps. In single-step prediction, we fed five measured pressure data points into the SVRM model to predict the next pressure data point. In multi-step prediction, we iterated the predicted pressure data points as part of the input data of the SVRM model for next prediction. The number of predicted data points used for next prediction was defined as the number of prediction steps. Multi-step predictions are desired to increase the time advance of the stall prediction. However, the prediction errors are accumulated in these multiple steps, and we must verify the maximum number of steps by which the SVRM model can predict the pressure data accurately enough to guarantee reliable stall prediction. In the experiments, we predicted the pressure data at 1–6 steps and demonstrate that the pressure data predicted by up to five steps are reliable for wavelet-transform-based stall prediction.

To further increase the time advance of stall prediction, we also increased the time interval of pressure data sampling by decreasing the sampling frequency from 320 Hz to 80 Hz. Since the rotational frequency of the centrifugal fan is 21.7 Hz, the new sampling frequency of 80 Hz still satisfies the sampling frequency theorem. The time-series pressure data were obtained from pressure sensor #2 at 80 Hz when the centrifugal fan was operated at 1300 rpm with guide vane openings of 0°, 15°, 30° and 45°. Two hundred and fisty pressure data points were obtained for each guide vane opening.

### 5.1. Single-Step Prediction

The time-series pressure data were first converted into their reconstructed phase space *X* = [*X*_1_, *X*_2_, …, *X_M_*] using Equation (1), which were then fed into the SVRM model to predict the pressure value of the next sampling point *Y*′ = *x*(*i* + 1 + (*m* − 1)*τ*. We generated the whole time series of the predicted pressure data using the measured pressure data, in which five measured pressure data points were used to predict the next data point. Using a personal computer (Pentinum Dual-Core CPU T4200@2.0 GHz; 2 GB RAM, Dell Computer, Round Rock, TEX, USA), the average computation time of the SVRM model was 0.00096 s.

**Figure 4.**
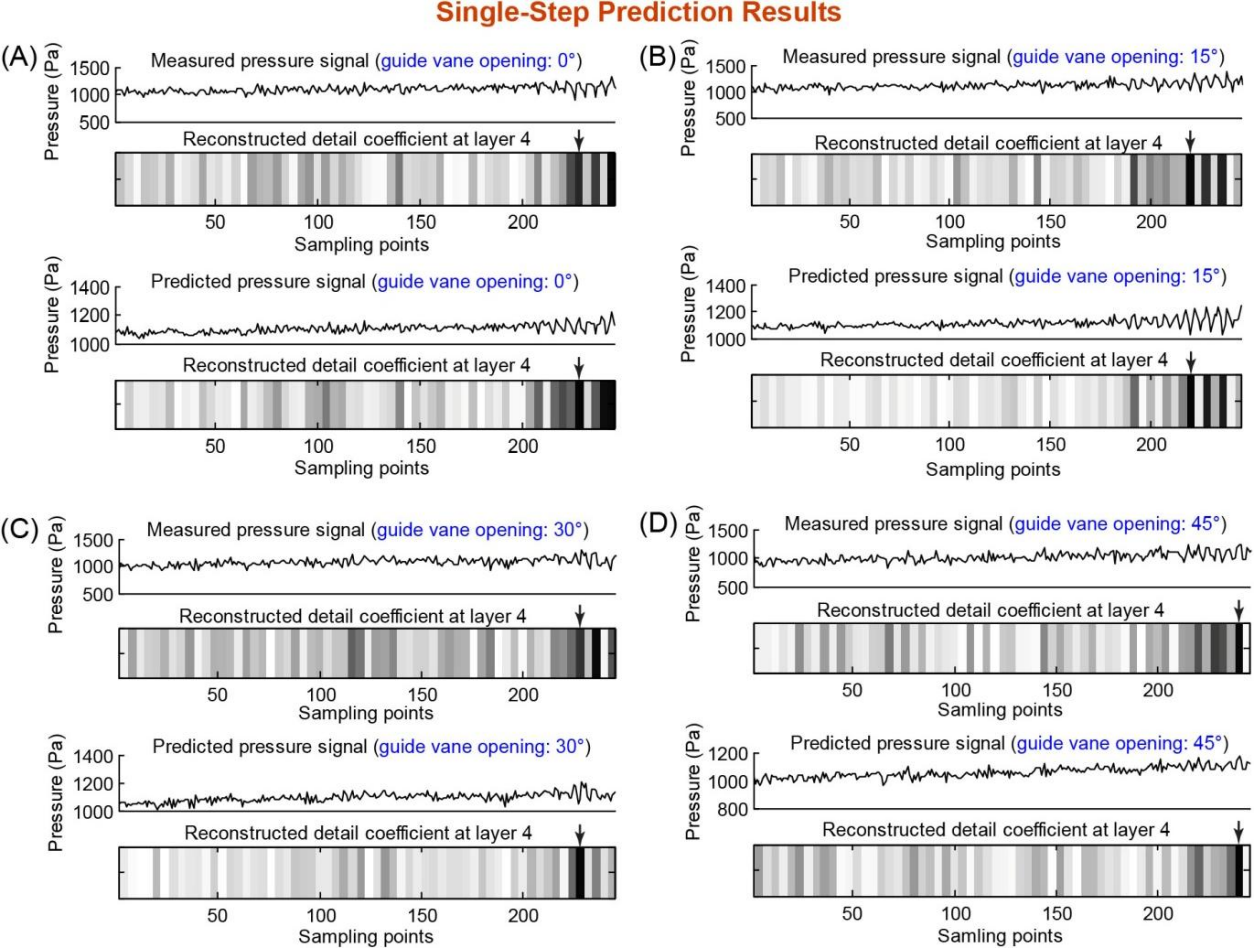
Single-step prediction results of the fan rotating stall at guide vane openings of 0°, 15°, 30° and 45° (panels (**A**)–(**D**)). In each panel ((**A**)–(**D**)), the experimental measured pressure signal and its wavelet analysis result (*i.e.*, fourth-layer detail coefficient) are shown on the top, and the SVRM-predicted pressure signal and its wavelet analysis result (*i.e.*, fourth-layer detail coefficient) are shown on the bottom. The arrows on the fourth-layer detail coefficient plot indicate the starting points of the rotation stall.

Figure 4 shows the following results: (i) the raw measured pressure data from sensor #2 (at guide vane openings of 0°, 15°, 30° and 45°); (ii) the predicted pressure data by the SVRM model; and (iii) the detail coefficient of the fourth layer whose frequency band (10–20 Hz) includes the rotating stall frequency (14.4 Hz) obtained through wavelet transform. We investigated the fourth-layer detail coefficient and used a threshold of 200 as the indicator of rotation stall, and the data points labelled with an arrow (in the four-layer detail coefficient plot) are the predicted starting point of the rotation stall. One can observe that the predicted pressure data sets (at all four guide vane openings) lead to the same prediction results (*i.e.*, the same starting points of rotation stall) as the measured pressure data sets. This demonstrates the feasibility of using our SVRM model for single-step rotation stall prediction.

### 5.2. Multi-Step Prediction

The time advance of rotation stall prediction is the most important parameter of any stall prediction technique. We determined the longest time advance of our SVRM prediction model by examining the maximum number of prediction steps the model can perform. Multi-step prediction was performed by iterating the previously predicted pressure data points as part of the input vector of the SVRM model. The experimental results show that, by up to five prediction steps, the SVRM model can predict pressure data accurate enough to yield reliable wavelet-transform-based stall prediction.

**Figure 5.**
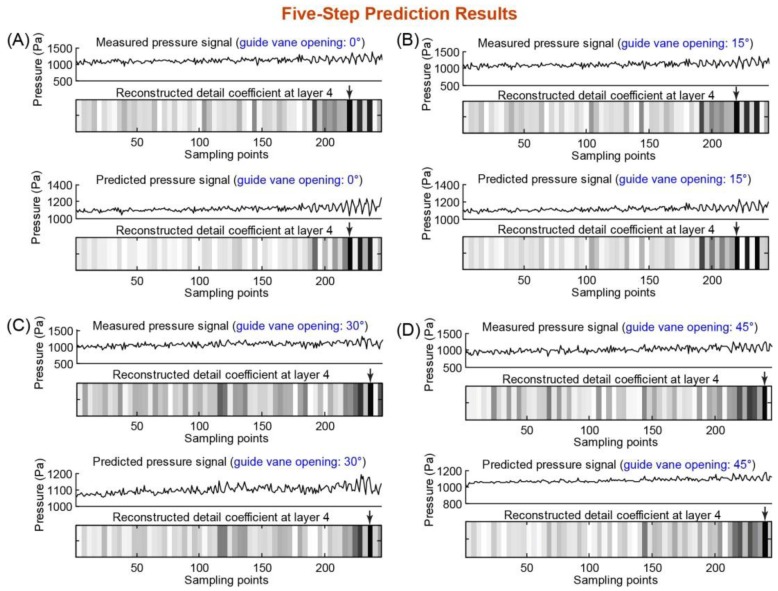
Five-step prediction results of the fan rotation stall at guide vane openings of 0°, 15°, 30° and 45° (panels (**A**)–(**D**)). In each panel ((**A**)–(**D**)), the experimental measured pressure signal and its wavelet analysis result (*i.e.*, fourth-layer detail coefficient) are shown on the top, and the SVRM-predicted pressure signal and its wavelet analysis result (*i.e.*, fourth-layer detail coefficient) are shown on the bottom. The arrows on the fourth-layer detail coefficient plot indicate the starting points of the rotation stall.

**Figure 6.**
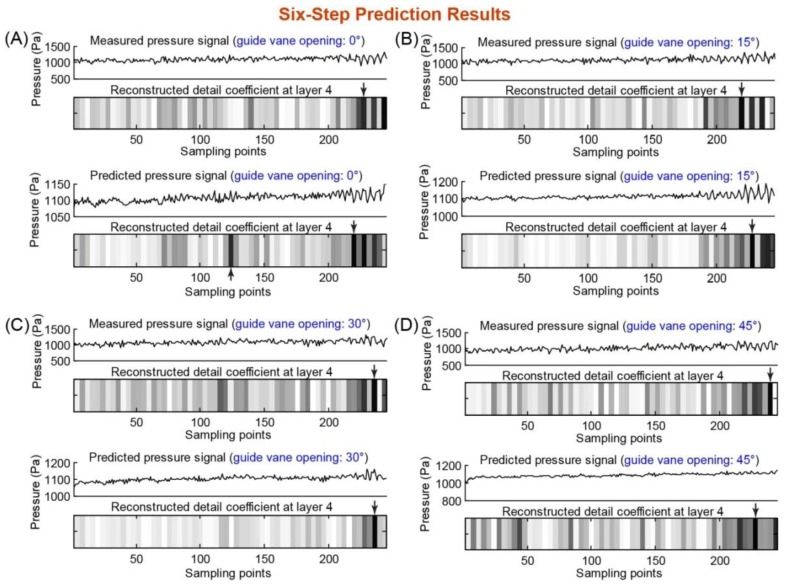
Six-step prediction results of the fan rotation stall at guide vane openings of 0°, 15°, 30° and 45° (panels (**A**)–(**D**)). In each panel ((**A**)–(**D**)), the experimental measured pressure signal and its wavelet analysis result (*i.e.*, fourth-layer detail coefficient) are shown on the top, and the SVRM-predicted pressure signal and its wavelet analysis result (*i.e.*, fourth-layer detail coefficient) are shown on the bottom. The arrows on the fourth-layer detail coefficient plot indicate the starting points of the rotation stall.

Figures 5 and 6 show the results of five- and six-step predictions. One can observe that the five-step prediction (Figure 5) accurately predicted the starting point of the rotation stall for all four guide vane openings (compared with the stall detection results of the measured pressure data). However, for the six-step prediction (Figure 6), the predicted pressure data did not yield an accurate prediction of the starting point of the rotation stall (the predicted and actual starting points of the rotation stall are not the same in Figure 6A,B,D), which was mainly due to the intolerable accumulation of multi-step prediction errors. The five-step advance means a prediction time advance of 0.0625 s, corresponding to approximately two rotor revolutions for axial compressors and 10 for industrial fans. This means they are, according to typical rotating frequencies, ranging from *O*(10^−3^ s) to *O*(10^−1^ s) [11]. Thus, the time advance of our prediction model is fairly significant and enough to allow a rotation stall control mechanism to react and prevent the stall development.

## 6. Conclusions

This paper presents the development and experimental validation of a SVRM model for intelligent prediction of rotation stalls of centrifugal fans in power plants. The internal pressure of the fan casing was used as the experimental data for rotation stall prediction. The SVRM prediction model was established using chaotic phase space transformation and a SVRM model, and was demonstrated to perform single and multi-step (up to five steps) prediction of the pressure data which can be further processed by real-time stall detection techniques. To validate the feasibility of using the SVRM model for rotation stall prediction, offline wavelet transform was performed for rotation stall prediction using both the measured and predicted pressure data sets. Through comparison of the stall prediction results obtained from the measured and predicted pressure data, we confirmed that the SVRM model is capable of predicting the pressure data by up to five steps and yielding reliable stall prediction. The effective time advance of stall prediction was 0.0625 s, which is significant enough for a stall control mechanism to react and suppress the stall development. The experimental studies were made on a 4-73No8D centrifugal fan, which is one of the most widely used centrifugal fans in Chinese power plants. We believe that this SVRM prediction model will find important applications in experimental rotation stall prediction and control of centrifugal and other types of fans, and therefore improve the power efficiency of combustion-based power plants.
